# What do we know about assessing healthcare students and professionals’ knowledge, attitude and practice regarding female genital mutilation? A systematic review

**DOI:** 10.1186/s12978-017-0318-1

**Published:** 2017-05-22

**Authors:** Jasmine Abdulcadir, Lale Say, Christina Pallitto

**Affiliations:** 1 Department of Obstetrics and Gynecology, Geneva University Hospitals, Faculty of Medicine, University of Geneva, 30 Bld de la Cluse, 1211 Geneva, Switzerland; 2 Department of Reproductive Health and Research, World Health Organization, 20, Avenue Appia, 1211 Geneva, Switzerland

**Keywords:** Female genital mutilation, Female genital cutting, FGM, Questionnaires, KAP, Knowledge, Attitude, Practice, Caregivers, Healthcare professionals

## Abstract

**Introduction:**

Improving healthcare providers’ capacities of prevention and treatment of female genital mutilation (FGM) is important given the fact that 200 million women and girls globally are living with FGM. However, training programs are lacking and often not evaluated. Validated and standardized tools to assess providers’ knowledge, attitude and practice (KAP) regarding FGM are lacking. Therefore, little evidence exists on the impact of training efforts on healthcare providers’ KAP on FGM. The aim of our paper is to systematically review the available published and grey literature on the existing quantitative tools (e.g. scales, questionnaires) measuring healthcare students’ and providers’ KAP on FGM.

**Main body:**

We systematically reviewed the published and grey literature on any quantitative assessment/measurement/evaluation of KAP of healthcare students and providers about FGM from January 1^st^, 1995 to July 12^th^, 2016. Twenty-nine papers met our inclusion criteria. We reviewed 18 full text questionnaires implemented and administered to healthcare professionals (students, nurses, midwives and physicians) in high and low income countries. The questionnaires assessed basic KAP on FGM. Some included personal and cultural beliefs, past clinical experiences, personal awareness of available clinical guidelines and laws, previous training on FGM, training needs, caregiver’s confidence in management of women with FGM, communication and personal perceptions. Identified gaps included the medical, psychological or surgical treatments indicated to improve girls and women’s health; correct diagnosis, recording ad reporting capacities; clitoral reconstruction and psychosexual care of circumcised women. Cultural and personal beliefs on FGM were investigated only in high prevalence countries. Few questionnaires addressed care of children, child protection strategies, treatment of short-term complications, and prevention.

**Conclusion:**

There is a need for implementation and testing of interventions aimed at improving healthcare professionals’ and students’ capacities of diagnosis, care and prevention of FGM. Designing tools for measuring the outcomes of such interventions is a critical aspect. A unique, reproducible and standardized questionnaire could be created to measure the effect of a particular training program. Such a tool would also allow comparisons between settings, countries and interventions. An ideal tool would test the clinical capacities of providers in managing complications and communicating with clients with FGM as well as changes in KAP.

## Plain English summary

Improving healthcare students and providers’ capacities of prevention and treatment of female genital mutilation (FGM) is important given the fact that 200 million women and girls globally are living with FGM. However, training programs and validated and standardized tools to assess providers’ knowledge, attitude and practice regarding FGM are lacking. Therefore, little evidence exists on the impact of training efforts on healthcare providers on FGM.

Our paper reviews the available literature on the existing quantitative tools (e.g. scales, questionnaires) measuring healthcare students’ and providers’ knowledge, attitude and practice on FGM. We reviewed 18 full text questionnaires implemented and administered to healthcare students’ and professionals in high and low income countries. The questionnaires assessed basic knowledge, attitude and practice on FGM. Some included personal and cultural beliefs, past clinical experiences, personal awareness of guidelines and laws, previous training on FGM, training needs, caregiver’s confidence in management of women with FGM, communication and personal perceptions. Identified gaps included the medical, psychological or surgical treatments indicated to improve girls and women’s health; correct diagnosis and psychosexual care of women who have undergone FGM. Cultural and personal beliefs on FGM were investigated only in high prevalence countries. Few questionnaires addressed care of children, child protection strategies, treatment of short-term complications, and prevention.

There is a need for implementation and testing of interventions aimed at improving healthcare students’ and professionals’ capacities of diagnosis, care and prevention of FGM. Designing a standardized tool for measuring the impact of interventions aimed at health care providers would generate evidence on what works to improve care for women and girls living with FGM.

## Background

Female Genital Mutilation (FGM) is not included in most of the pre- and post-graduate curricula of health care providers [1]. The new guidelines of the World Health Organization (WHO) on the management of FGM complications recommend that healthcare providers be trained so that they are able to offer evidence based and respectful information, health education and care to girls and women living with FGM [2]. According to WHO, training could also increase prevention capacities and decrease the “medicalization” of FGM, which is defined as genital cutting by a healthcare provider in any setting and at any point in a woman’s life [2].

Healthcare professionals have a key role in providing informed care, which includes identifying and treating psychological and physical health consequences of FGM; as well as in recording the practice in medical files, reporting it to authorities where appropriate and preventing the practice from being carried out. Health care providers often receive little to no training on how to provide care and treatment to women and girls with FGM, and when training does occur [3], the clinical, epidemiological and legal impact of training on healthcare professionals’ knowledge, practice and attitude is rarely assessed. A recent systematic review of the published and grey literature on the interventions aimed at improving healthcare providers’ capacities of prevention and treatment of FGM found only two studies reporting improvement in knowledge of the practice and confidence in treating it among participants [4], showing the lack of evidence on existing training programs [5].

The aim of our paper is to systematically review the available published and grey literature on the existing quantitative tools (e.g. scales, questionnaires) measuring healthcare students’ and providers’ knowledge, attitude and practice (KAP) regarding FGM. The paper will summarize the available existing tools, discuss gaps in evidence and will inform the development of a unique, standardized, comprehensive instrument to evaluate training interventions in low and high income countries.

## Methods

The present systematic review was conducted following the PRISMA (Preferred Reporting Items for Systematic reviews and meta-Analyses) guidelines [6]. The available published and grey literature on assessing healthcare students and providers’ knowledge, attitude and practice regarding female genital mutilation was identified using a protocol designed for this purpose. The protocol is available on request. The systematic search included two online databases: Pubmed/Medline and Popline. We considered publications from January 1^st^, 1995 to July 12^th^, 2016. The keywords used in the search were “female genital mutilation”, “female genital cutting”, “female circumcision”, “infibulation”, “excision”, “female genital mutilation/cutting”, “health personnel”, “healthcare providers”, “nurses”, “midwives”, “doctors”, “community health workers”, “physicians”, “education”, “training”, “guidelines”, “knowledge”, “attitude”, “intervention”, “practice”, “questionnaire”, “interview”, “survey”. The terms were used in various combinations. Hand searching was performed through the reference list of the relevant papers. When the actual tools used to assess knowledge, practice and attitude were not available in the papers, the authors were contacted by email to request a copy.

We had no language restriction and included any study design that reported any quantitative assessment/measurement/evaluation of knowledge, attitude and practice of healthcare providers about FGM. To ensure a comprehensive overview of existing tools and to inform the development of a future instrument, we searched for relevant papers published during the past 21 years in Pubmed and Popline.

JA retrieved, screened the studies for relevance to the research question and extracted data from the included studies.

## Results

**Fig. 1:**
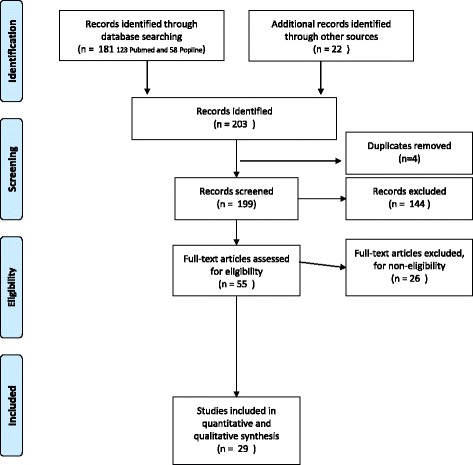
PRISMA 2009 flow diagram

Our search yielded 199 publications eligible for screening. After title and abstract screening, 55 articles were included for full text review. Twenty-nine of these met our inclusion criteria (Fig. 1). We had access to the full text of the quantitative assessment tools employed to measure knowledge, attitude and practice of healthcare students and professionals in 19 of these papers (18 distinct questionnaires as two of the papers presented the same tool) (Table 1) [7, 8]. In spite of multiple efforts to locate the remaining questionnaires, we were unable to retrieve them. Of the tools we could not access, many were from high prevalence African countries: Egypt [9–14], Mali [15], Nigeria [16, 17] and Sudan [18]. The remaining two unavailable tools had been administered in Sweden [19] and UK [20]. The available questionnaires we fully reviewed were from the UK [21–23], Australia [24, 25], Belgium [26, 27], Switzerland [28, 29], the U.S. [30, 31], Spain [32], Sweden [33], the Gambia (same questionnaire in two included studies) [7, 8], Nigeria [34] and Sudan [35].

**Table 1:** Information on the questionnaires reviewed

Authors	Country	Items evaluated	Number of Participants	Caregivers categories	Tool employed	Availability of the tool	Development of the tool	Interests	Gaps
1)Moeed 2012	Australia	Experience	530	Fellows, diplomats and trainees provided by Royal Australian and New Zealand College of Obstetricians and Gynecologists	Questionnaire: 7 questions	Yes	-	Evaluation of experience of reinfibulation	Basic assessment of previous encounters with women with FGM and theoretical knowledge. No evaluation of clinical knowledge and management of FGM, prevention and law
2)Sureshkumar 2016	Australia	Knowledge, attitude, experience, awareness of clinical guidelines, education/training needs	497	Pediatricians	Questionnaire: 31 questions	Yes	Working group of expert clinicians (general pediatrics, child protection, obstetrics and gynecology) and the founder of African Women Australia. Questionnaire piloted for acceptability, content and clarity by ten pediatricians from different specialties and amended according to feedback	Evaluation of awareness/knowledge of guidelines	Basic evaluation of previous experience, awareness and theoretical knowledge on FGM. No evaluation of clinical knowledge and management of FGM, prevention and law
3)Leye 2008	Belgium	Knowledge, attitude and practice	334	Gynecologists	Questionnaire: 26 questions	Yes	Based on a similar survey conducted in Switzerland among midwives, gynecologists, pediatricians and social services	Evaluation of awareness/knowledge of guidelines. Evaluation of knowledge and attitude on reinfibulation, cosmetic surgery, piercing and pricking. Assessment of experience of medicalization	Basic evaluation of previous experience, awareness and theoretical knowledge on FGM. No evaluation of clinical knowledge and management of FGM, prevention
4)Cappon 2015	Belgium	Knowledge, attitude and practice	820	Midwives	Questionnaire: 23 questions	Yes	Adapted from a survey conducted among Flemish gynecologists. Pilot study among midwives who were not part of the study population. Their feedback was integrated in the final version of the questionnaire	Evaluation of awareness/knowledge of guidelines. Evaluation of knowledge and attitude on reinfibulation, cosmetic surgery, piercing and pricking. Assessment of experience of medicalization	Basic evaluation of previous experience, awareness and theoretical knowledge on FGM. No evaluation of clinical knowledge and management of FGM, prevention
5)Kaplan 2009	Spain	Knowledge, attitude and practice	225 in 2001 and 184 in 2004	Family physicians, pediatricians, nurses, midwives and gynecologists	Questionnaire: 9 questions	Yes	-	-	Basic assessment of previous encounters with women with FGM and theoretical knowledge. No evaluation of clinical knowledge and management of FGM, prevention and law
6)Tamaddon 2006	Sweden	Knowledge, experience	769	Midwives, gynecologists, pediatricians. Hospital, youth clinics, maternal health clinics, school physicians and nurses	Questionnaire: 10 questions	Yes	Informally preview of the questionnaire by a few midwives and gynecologists, a pediatrician, and a school nurse, and then improved before it was sent out to the survey group	-	Basic evaluation of previous experience with patients with FGM. No objective assessment of knowledge, attitude and practice
7)UNICEF 2012	Switzerland	Knowledge and practice. Knowledge of guidelines	1053	Obstetricians, gynecologists, midwives and asylum personnel	Questionnaire: 21 questions	Yes	Same survey in 2001, 2004	Assessment of availability of certified interpreters	Basic assessment of previous encounters with women with FGM and theoretical knowledge. No evaluation of clinical knowledge and management of FGM, prevention and law
8) UNICEF 2016	Switzerland	Knowledge and practice	75	Obstetricians, gynecologists, midwives and asylum personnel	Questionnaire: 33 questions	Yes	-	Assessment of awareness of asylum rights based on FGM	Basic assessment of previous encounters with women with FGM and theoretical knowledge. No evaluation of clinical knowledge and management of FGM, prevention and law
9)Elliott 2016	UK	Knowledge and attitude	49	Psychosexual therapists	Questionnaire: 21 questions	Yes	Developed from an earlier and briefer version	Evaluation of knowledge and attitude on reinfibulation, cosmetic surgery among men and women, piercing and pricking, male circumcision, parents legal responsibility	Basic assessment of theoretical knowledge. No evaluation of clinical knowledge and management of FGM, prevention and law
10)Relph 2013	UK	Knowledge, attitude and training	79	Obstetricians, pediatricians, midwives, student midwives, foundation year trainees and medical students	Questionnaire: 19 questions	Yes	Based on the Royal College of Obstetrics and Gynecology guidelines and on similar questionnaires by authors from Belgium and Egypt. Small pilot study on 5 participants (medical students, junior doctors and a consultant in obstetrics and gynecology)	Evaluation of attitude includes medicalization, age of consent, reinfibulation, cosmetic surgery and genital piercing, male circumcision	Basic assessment of clinical and theoretical knowledge on FGM, defibulation, law. No evaluation of clinical knowledge and management of FGM, prevention and law
11)Purchase 2013	UK	Knowledge of guidelines	607	Fellows and trainees of the Royal College of Obstetricians and Gynecologists	Questionnaire: 19 questions	Yes	On-line survey based on the Royal College of Obstetrics and Gynecology guidelines	Knowledge score. Questions on communication	Basic assessment of clinical and theoretical knowledge on FGM, defibulation, law, referral. No evaluation of clinical knowledge and management of FGM, prevention and law
12)Jacoby 2013	U.S.	Confidence in management of FGM type III	11	Midwives	Questionnaire: 9 questions	Yes	Evaluation of a training course with 9 objectives, The participants were asked to rate these objectives using a 5-point Likert scale ranging from no confidence to very confident	Evaluation of confidence in counseling women before defibulation, recording FGM	Basic assessment of confidence in management of women with FGM type III. No evaluation of clinical knowledge and management of FGM, prevention and law
13)Hess 2010	U.S.	Knowledge and experience	243	Midwives and nurses members of the American College of Nurse-Midwives	Questionnaire: 13 questions	Yes	Review by 3 nurses who had experience in women’s health and with clients with a history of FGM. No pilot study before distribution of the survey	Evaluation of knowledge about cultural beliefs on FGM and stigmatization of migrant circumcised women. Assessment of cesarean section indication because of FGM	Basic assessment of theoretical knowledge on FGM and law. No evaluation of clinical knowledge and management of FGM, prevention and law
14)Kaplan 2013	Gambia	Knowledge, attitude and practice	468	Nurses and midwives	Questionnaire: 36 questions	Yes	Pilot study conducted in 2 regions of the country among 97 caregivers. Designed by a medical anthropologist researcher with a thorough and extensive ethnographic background in The Gambia, following the implementation of barrier analysis using focus group discussions among Gambian men and women of all ethnic groups	Evaluation of alternative initiation rites to FGM. Information on daughter’s circumcision, existence of FGM in the own family, opinions on how to stop FGM	Basic assessment of theoretical knowledge on FGM and law. No evaluation of clinical knowledge and management of FGM, prevention and law
15)Kaplan 2016	Gambia	Knowledge, attitude and practice	1288	Nurses, midwives, public health officers, students of medicine, nursing, midwifery, and public health degrees	Questionnaire: 36 questions	Yes	Pilot study conducted in 2 regions of the country	Evaluation of alternative initiation rites to FGM. Information on daughter’s circumcision, existence of FGM in the own family, opinions on how to stop FGM	Basic assessment of theoretical knowledge on FGM and law. No evaluation of clinical knowledge and management of FGM, prevention and law
16)Onuh 2006	Nigeria	Knowledge, attitude, practice	182	Nurses	Questionnaire: 20 questions	Yes	Pretest among 24 nurses by a pilot stu[1–3]dy	Information collected included ethnic group, religion, being circumcised, daughter’s circumcision, medicalization and traditional myths	Basic assessment of clinical and theoretical knowledge on FGM. No evaluation of clinical knowledge and management of FGM, prevention and law
18)UNFPA 2016	Sudan	Knowledge, attitude and perception	308	Midwives	Questionnaire: 29 questions	Yes	-	-	Basic assessment of previous encounters with women with FGM and theoretical knowledge. No evaluation of clinical knowledge and management of FGM, prevention and law
19)Widmark 2002	Sweden	Knowledge, attitude, practice, emotions, communication	26	Midwives	Questionnaire (associated to interviews and focus groups)	No	A Kenyan sociologist, a Swedish midwife and a Somali physician	-	-
20)Zaidi 2007	UK	Knowledge, experience and practice	45	Midwives, Obstetricians	Questionnaire	No	2 senior clinicians, based on recommendations of the Royal College of Obstetrics and Gynecology	-	-
21) Refaat 2009	Egypt	Determinants of medicalization of FGM	193	Physicians	Questionnaire: 72 questions	No	Based on a conceptual model hypothesized by the author whereby physicians may be practicing FGM due to: cultural influences, financial benefits, or lack of knowledge about the consequences	-	-
22)Refaat 2007	Egypt	Knowledge and attitude	50	Recently graduated physicians	Questionnaire	No	-	-	-
23)Rasheed 2011	Egypt	Attitude	801	Nurses, young and senior pediatricians and gynecologists	Questionnaire	No	-	-	-
24)Mostafa 2006	Egypt	Knowledge, attitude, beliefs	330	Medical students (5^th^ year)	Questionnaire	No	Based on literature review. Revision by 3 community medicine professors	-	-
25) Allam 1999 26)Allam [4, 5]2001	Egypt	Knowledge and beliefs	1070	University students	Questionnaire: 32 questions	No	-	Evaluation of information sources	-
27)Newman 2003	Mali	Knowledge, skills and awareness	120	Reproductive health providers	Questionnaire	No	Developed to assess a national FGM curriculum	-	-
28)Adekanle 2011	Nigeria	Knowledge and experience	250	Physicians, nurses, midwives	Questionnaire	No	Based on the government passage of bill against FGM	-	-
29)Ashimi 2014	Nigeria	Knowledge and attitude	350	Nurses	Questionnaire	No	Pre-tested	-	-
30)Ali 2012	Sudan	Knowledge and attitude	157	Midwives and TBA	Questionnaire	No	-	-	-

The questionnaires identified focused on basic assessment of knowledge, attitude and practice regarding FGM. Subjects included existing types of FGM, main physical complications, knowledge of countries and religions where FGM is practiced, cultural reasons for genital cutting, defibulation, obstetric care in the event of FGM and reinfibulation.

Some tools enquired about past clinical experiences and encounters with women with FGM, personal awareness of available clinical guidelines and local laws, previous training on FGM, training needs, caregiver’s confidence in clinical management of women with FGM, communication with clients and personal perceptions when caring for women living with FGM.

Healthcare providers’ feelings about women with FGM were investigated by only one questionnaire, which was administered to midwives in Sweden (full text unavailable for the review) [19]. The personal and cultural beliefs on FGM and the presence of genital cutting in one’s own family were only asked in studies conducted in African countries, where FGM was considered a traditional practice. Cultural beliefs of healthcare professionals regarding women with FGM were not assessed in high income countries.

Determinants of medicalization were investigated in both high and low income countries. In the UK and in Belgium, some specific topics covered included highly debated subjects such as medical and legal controversies regarding FGM and cosmetic surgery, appropriate terminology to use when speaking to a woman affected by FGM, personal opinions concerning pricking and piercing classified as FGM type IV and differences between FGM and male circumcision [21, 22, 26, 27].

The questionnaires used in Switzerland asked caregivers about the availability of certified interpreters when caring for women with FGM and on awareness of asylum rights related to genital cutting [28, 29].

Kaplan and colleagues’ conducting research in the Gambia asked healthcare professionals about their attitude concerning alternative initiation rites and discrimination of uncut girls/women [7, 8].

The areas of knowledge, attitude and practice identified in the different questionnaires are illustrated in Table 2.

**Table 2:** Synthesis of the areas of knowledge, attitude and practice explored by the 18 full questionnaires reviewed

Authors	Country	Experience and practice					Attitude							Knowledge					
		Previous clinical encounter with women with FGM	Asking women about FGM	Previous request of performing reinfibulation or FGM. Knowledge of FGM happening overseas	Performing defibulation	Other practices^a^	Human rights	Health risks	FGM practice^b^	Training regarding FGM^c^	Current controversial issues^d^	Child protection, reporting and law against FGM	Inclusion of men when discussing about FGM	Information on FGM^e^	Guidelines and other available resources	FGM complications	Child protection and law	Pricking and FGCS	Management^f^
Moeed 2012	Australia	x	x	x															
Sureshkumar 2016	Australia	x	x	x	x			x	x		x				x	x	x		
Leye 2008	Belgium	x	x	x	x		x	x		x	x	x	x		x	x	x	x	x
Cappon 2015	Belgium	x	x	x	x	x		x		x		x			x	x		x	x
Kaplan 2009	Spain	x			x					x	x				x	x			
Tamaddon 2006	Sweden	x	x	x							x								
UNICEF 2012	Switzerland	x	x	x	x	x	x			x	x								
UNICEF 2016	Switzerland						x									x		x	
Elliott 2016	UK									x		x			x		x	x	x
Relph 2013	UK	x		x	x					x	x	x	x		x		x	x	
Purchase 2013	UK	x			x		x			x	x	x					x	x	
Jacoby 2013	U.S.A										x								
Hess 2010	U.S.A														x		x	x	
Kaplan 2013 and 2016	Gambia					x		x		x		x	x	x	x		x	x	
Onuh 2006	Nigeria									x			x		x		x		
UNFPA 2016	Sudan	x	x		x	x			x	x				x	x		x		

^a^Other practices include previous experiences and practices related to asylum and FGM, risk assessment and protection of girls, working with certified interpreters, availability of specialized care, specific local services; referral centers and local guidelines

^b^Attitude on FGM practice includes attitude on the rite of passage of FGM; women with and without FGM, including difficulties when caring for/discussing with women with FGM, reinfibulation and defibulation; parents who allow FGM; medicalization; FGM for a own daughter or in the own family; role of caregivers; reasons for performing FGM

^c^Training regarding FGM includes interest in further training; opinion on available training; self confidence on self knowledge on FGM

^d^Controversial issues are pricking, female genital cosmetic surgeries, piercing, male circumcision, reinfibulation, FGM requested by adult women, terminology and alternative rituals to FGM

^e^Information on FGM including religion of communities practicing it, countries at risk, prevalence, definition, classification, reasons of the practice, stigmatization in host country

^f^Management of complications of FGM, in particular type III. Defibulation. C-section in case of FGM

The questionnaires identified in the present systematic review were administered to a range of populations, including university students, nurses, midwives and physicians (general practitioners, obstetricians and gynecologists, pediatricians and fellows in these specializations). Psychosexual therapists were included only in UK [21], social workers and asylum personnel in Switzerland, public health workers in the Gambia [8, 28, 29] and traditional birth attendants in Sudan (full text questionnaire unavailable) [18].

The number of respondents varied from 11 [31] to 1288 people [8]. The length of the assessment tools ranged from 7 [24] to 36 questions [7, 8]. One unavailable questionnaire focusing on medicalization of FGM in Egypt included 72 questions [14]. Response options included multiple choice, free text or Likert scales.

The questionnaires reviewed were generally developed by experts in FGM and were based on local clinical recommendations/guidelines on FGM. In agreement with such resources, the main expectations regarding KAP of the healthcare professionals interviewed were for instance not performing C-section in case of FGM type III; offering defibulation to treat or avoid urogynecological, sexual and obstetric complications; being against reinfibulation and any form of medicalization; being aware that FGM is illegal, a human rights violation and a dangerous practice responsible for several short and long term complications. Some of the questionnaires were informally piloted or pre-tested for acceptability, content, clarity and feedback in small groups of caregivers or students [7, 8, 17, 23, 25, 26, 33, 34]. Only two questionnaires, implemented in Belgium and U.K., were based on previous questionnaires used in another country [23, 27]. The questionnaires were administered in person, generally by medical students or midwives trained to administer the questionnaire, or by email or postal mail.

Only in Switzerland, Belgium and the Gambia the questionnaires were administered more than once [7, 8, 26–29]. Two papers reported administering the same instrument to assess an improvement of knowledge, attitude and practice after a training intervention [21, 31].

## Discussion

Several questionnaires have been developed and used in low/middle (LMIC) and high income countries (HIC) to assess knowledge, attitude, experience, beliefs and practice of healthcare professionals regarding FGM. The main contents of these questionnaires were similar regardless of setting (LMIC and HIC), which included high prevalence countries in Africa and countries of the diaspora. No studies of this kind were conducted in other high prevalence countries outside of Africa, such as Indonesia and Malaysia.

The main gap identified in the questionnaires, which reflects the gap in training, relates to the assessment of clinical management of women with FGM to prevent and treat complications. Questionnaires tended to enquire about existing types and complications of FGM rather than about the medical, psychological or surgical treatments indicated to improve girls and women’s health. Knowledge of defibulation, in particular during pregnancy, as a specific treatment option was assessed in some questionnaires. There was little assessment of the accuracy of diagnosis, recording capacities, clitoral reconstruction and psychosexual care of affected women.

Cultural and personal beliefs on cut and uncut women were investigated only in high prevalence countries and never in high income countries despite the fact that providers in host countries may also hold false beliefs that could negatively affect healthcare. The care of children with FGM who present with short term complications were not investigated in most of the questionnaires. Knowledge or actions around child protection strategies were also rarely assessed. In addition, there was little attention to prevention of FGM and about potential strategies that health care providers could take to prevent FGM.

Although several questionnaires exist, authors tended to develop new measurement tools, which they did not use more than once in most of the settings. With the exception of two questionnaires, all of the instruments were used to assess caregivers’ knowledge, attitude and practice. Only in U.S. and U.K. were questionnaires administered at baseline and after a training intervention to evaluate a potential improvement among the health care providers [21, 31]. Assessing healthcare providers’ KAP is different than evaluating learning, learning transfer and the impact of a training, in particular on practice and in the long term. In addition, KAP assessments should be associated with or followed by health professionals support and training.

The review of the instruments revealed that there were no comparisons made between different populations of caregivers, settings and countries. LMIC and HIC providers could differ in KAP, cultural and traditional views and available resources. This implies differences in training and support needs. One of the examples is the FGM “medicalization” in high prevalence countries such as Egypt and Sudan, which has been explained by social pressure, the belief that a medicalized genital cutting reduces harm and dangers, financial reasons or lack of laws forbidding FGM [36]. Training on when to perform defibulation during pregnancy or labour might also be adapted depending on women’s patterns of accessing care for antenatal care and during labour depending on the setting.

Statistical analyses to explore potential associations or correlations between the training of caregivers and their clinical practice or attitude were not conducted in any of the studies. Therefore, there is a lack of rigorous evidence on the impact of existing training programs on actual practice.

## Conclusion

The review confirmed the need for education and training of healthcare professionals [1, 37], who are key persons in the care of complications and prevention of FGM. Interventions aimed at improving health providers’ knowledge, skills, attitude, communication and prevention have been developed and implemented worldwide [3], but these often vary in quality and content. Furthermore, the effect of these trainings on clinical practice is unknown because of the lack of rigorous evaluation of acceptability and efficacy. A unique, validated and reproducible questionnaire to measure the impact of training interventions would facilitate the evaluation of future training and would also allow comparisons between different settings, interventions and countries. Such a tool could be progressively improved based on the experiences during its use. A standardized tool should test the real clinical and communication capacities of providers in managing complications of clients with FGM, while also assessing other aspects of care including attitude, practice, experience, prevention and legal considerations. Designing a standardized tool for measuring the outcomes of such interventions would increase evidence on existing programs, which would help to improve these programs and ultimately improve care of the millions of women and girls living with FGM.

## Abbreviations

FGM: Female genital mutilation
TBA: Traditional birth attendants
WHO: World Health Organization
